# Carbonated magmatic sulfide systems: Still or sparkling?

**DOI:** 10.1126/sciadv.adl3127

**Published:** 2024-06-21

**Authors:** Maria Cherdantseva, Michael Anenburg, Marco Fiorentini, John Mavrogenes

**Affiliations:** ^1^ Centre for Exploration Targeting, School of Earth Sciences, The University of Western Australia, 35 Stirling Hwy, 6009 Crawley, Perth, WA, Australia.; ^2^ Research School of Earth Sciences, Australian National University, Canberra, ACT 2600, Australia.

## Abstract

For decades, there has been debate surrounding the transport of dense metal-rich sulfide liquid in mafic magmas. This topic is crucial to understanding the genesis of valuable resources of nickel, copper, and platinum-group elements, which are essential for a sustainable, emission-free energy future. Recent studies of mineralized mafic magmas suggested that gas bubbles adhere to sulfide globules, reducing their density and favoring upward transport. While this hypothesis may explain sulfide mobility in near-surface magmatic environments, it is at odds with key mineralogic and textural observations and does not explain how long-distance sulfide transport operates. Here, we suggest an alternative hypothesis that builds on previous observations, focusing on the interaction between carbonate melt and sulfide liquid. We demonstrate experimentally that carbonate melt wraps sulfide globules forming a relatively mobile pair that may mediate metal-rich sulfide transport from mantle to crust.

## Teaser

Immiscible carbonate melt emerges as a crucial buoyancy aid for metal-rich sulfide liquids in mafic-ultramafic magmas.

## INTRODUCTION

Understanding the intricate mechanisms responsible for the transport of critical metals, including nickel, copper, and platinum-group elements (PGEs), from Earth’s mantle to crustal reservoirs holds paramount impact. These resources play a pivotal role in driving the transition from conventional fossil fuels to environmentally friendly “green” energy sources. Furthermore, they underpin the advancement of contemporary technologies encompassing communication, computing, health care, and aerospace while also underwriting the robustness of national security frameworks.

Empirical evidence firmly establishes the central role of mantle-derived magmas in transporting these vital metals. These magmas exhibit varying levels of alkalinity and are characterized by substantial MgO contents, generally exceeding 8 wt % (*1*). Upon sulfide saturation, these metal-rich magmas exsolve an immiscible sulfide liquid, which concentrates metals from the silicate melt, forming—if other conditions are met—a magmatic sulfide deposit (*2*–*4*). However, it is known that sulfur solubility in mantle-derived melts displays an inverse correlation with pressure (*5*, *6*) such that ascending magmas become increasingly sulfide undersaturated and sulfide liquids dissolve into the silicate magma, unless they undergo crustal assimilation of sulfur-bearing materials (*7*) or extreme fractionation of silicate minerals (*8*, *9*).

Recent studies have presented alternative models that emphasize the importance of mechanical incorporation of metal-bearing sulfide from deep sources (*10*–*12*). If voluminous metal-rich sulfide liquids or nanomelts could be transported directly from the mantle, requirements of crustal assimilation or extreme fractionation to trigger sulfide saturation would not be necessary. These changes to the conventional model would affect the way we explore for magmatic sulfide deposits, as geochemical proxies designed to fingerprint crustal contamination of mafic magmas may not be essential (*13*, *14*).

Whereas microdroplets, nanomelts, and nuggets can be easily transported in magma due to their size, the mechanisms facilitating the efficient transport of large droplets of dense, metal-rich sulfide liquids within nonviscous silicate magmas continue to be a topic of debate. Given the substantial density contrast (*15*) and large surface tension at the interface between sulfide and silicate liquids (*16*, *17*), the former tend to be stranded within crystal mushes (*18*, *19*). Therefore, achieving extended vertical transport of sulfide liquid over large distances requires one or more additional mechanism(s).

One of the models proposed to address this issue focuses on the role of gas bubbles and their physical affinity to sulfides, inspired by metallurgical practices where metals are attached to gas bubbles during flotation (*20*). Experimental evidence demonstrates that sulfide globules could adhere to the bottoms of gas bubbles within silicate magmas, maintaining this attachment due to the dominance of capillary forces over buoyancy forces (*21*). Subsequently, these experimental findings were extended to natural mineral systems, as sulfide minerals in several magmatic sulfide ore deposits exhibit spatial associations with volatile-rich phases, such as apatite, phlogopite, amphibole, and calcite (*22*, *23*).

On the basis of the rounded morphologies of rock domains containing volatile-rich minerals, they were interpreted as remnants of gas bubbles attached to sulfide globules and later filled with either highly fractionated magma or secondary phases (*10*, *21*, *24*, *25*). This concept triggered a cascade of subsequent studies and gained increasing popularity (*26*–*28*), supporting previous studies on the potential role of volatiles in metal transport (*29*–*33*). The adherence of sulfide globules to gas bubbles may enhance their transport, especially in shallow magmatic-hydrothermal systems (*34*); however, the extension of this “bubble” model of sulfide mobilization to deep systems (*10*, *11*), where gas bubbles are unlikely to exist due to their high solubilities at high pressures, might not be suitable.

The aim of the current study is to identify an alternative explanation for the rounded halos of volatile- and incompatible element–rich minerals associated with magmatic sulfide globules. Analogous textures and mineralogical assemblages surrounding sulfides are documented in several deposits emplaced at variable crustal levels, including the lower crust where gas-driven sulfide transport is not ubiquitously applicable given the high-pressure conditions. Hence, an alternative carrier that can aid the transport of sulfide liquid across the lithosphere must be identified. Here, we propose, based on a series of experimental and natural observations, that carbonate melt–wrapped sulfide globules are a better option than the previously proposed gas bubbles to aid dense sulfide liquid on its journey across the lithosphere.

### Compound globules

**Fig. 1.**
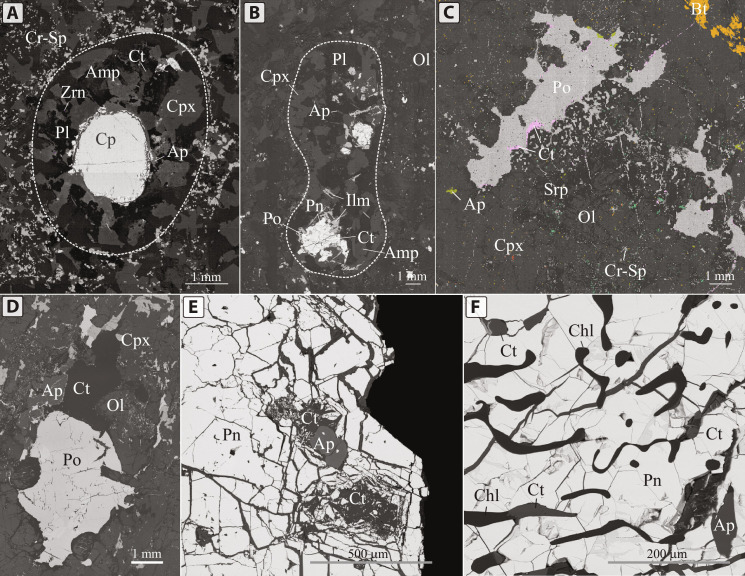
Backscattered electron images of sulfide globules associated with volatile-rich phases from several locations. (**A**) Sulfide globule surrounded by a volatile-rich halo from the Norilsk 1 intrusion, Russia (*35*). (**B**) Large elongate halo from the Rudniy intrusion, Mongolia. (**C**) Sulfide globule from the Valmaggia pipe, Italy, rimmed by calcite, surrounded by an assemblage of volatile- and incompatible element–rich phases. (**D**) Calcite and apatite attached to a small sulfide globule from the Valmaggia pipe, Italy. (**E**) Sulfide globule from the Rudniy intrusion, Mongolia, containing irregularly shaped calcite and apatite inclusions. (**F**) Sulfide globule containing vermicular apatite and calcite inclusions. Ap, apatite; Chl, chlorite; Ct, calcite; Cp, chalcopyrite; Cpx, clinopyroxene; Cr-Sp, chromian spinel; Ilm, ilmenite; Ol, olivine; Pl, plagioclase; Pn, pentlandite; Po, pyrrhotite.

The compound globules documented in this study are a combination of two globular assemblages hosted in mafic-ultramafic intrusions: a sulfide globule in the middle and a volatile- and incompatible element–rich assemblage around it (Fig. 1, A and B). These compound globules are common in some globular sulfide ores but are not found in all magmatic sulfide deposits worldwide. Below, we provide a compilation of the main features of compound globules described in the literature.

Compound globules were described in detail in several deposits and mineralized mafic intrusions, such as the giant upper crustal Norilsk deposit (Fig. 1A) (*25*, *35*), the upper crustal Rudniy intrusion in northwest Mongolia (Fig. 1B) (*36*), and others. These mineralized intrusions, composed of gabbro, gabbronorite, and troctolite, generally preserve evidence of rapid crystallization, such as elongated skeletal crystals of olivine, plagioclase, and chromian spinel (*25*, *36*). In many cases, the volatile- and incompatible element–rich halos around sulfide globules are not rounded in shape yet still preserve the same mineralogical assemblage in spatial association with magmatic sulfide minerals. Examples of these intrusions are the lower crustal Valmaggia pipe in northern Italy (Fig. 1C) (*29*, *37*), the Stillwater Complex in the United States (*22*), the mid-crustal Bushveld deposit in South Africa (*38*), and the Jinchuan deposit in China (*39*). These deposits are of different locations, sources, ages, and emplacement conditions, yet contain evidence of compound globules, preserving similar textures and mineral assemblages in halos around sulfide globules.

**Fig. 2.**
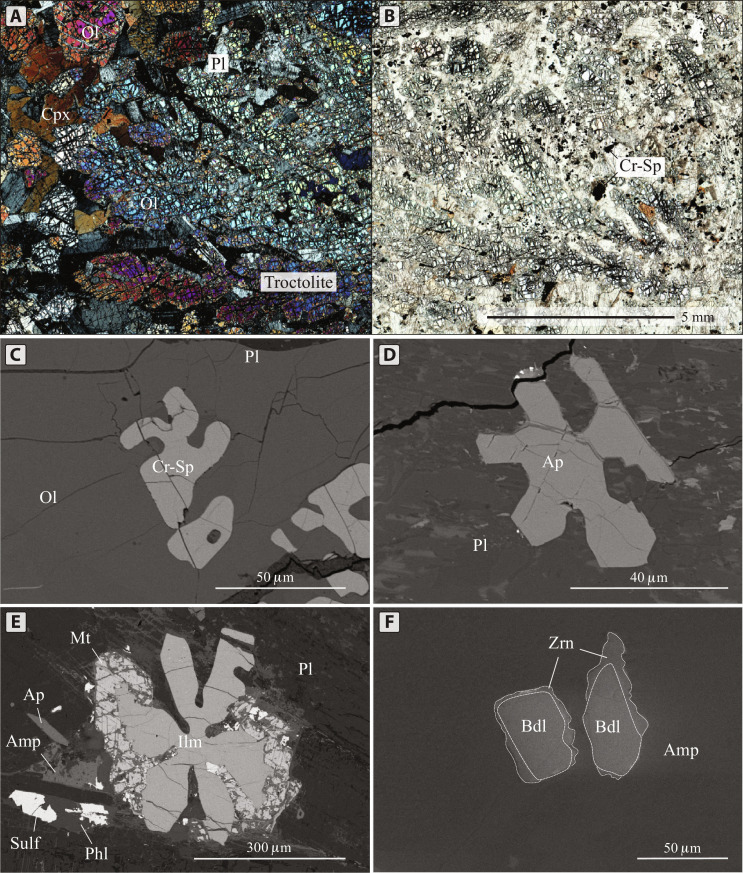
Images of skeletal elongated crystals of olivine and dendritic crystals of chromian spinel in troctolite, as well as skeletal crystals of ilmenite and apatite and zoned crystals of baddeleyite from volatile- and incompatible element–rich halos surrounding sulfide globules. (**A** and **B**) Transmitted light microphotograph of dendritic crystals of olivine in troctolite in (A) crossed and (B) plain polarized light. (**C** to **E**) Backscattered electron (BSE) images demonstrating (C) a dendritic crystal of chromian spinel in troctolite, (D) skeletal dendritic crystals of apatite inside the halo, and (E) a skeletal crystal of ilmenite from the halo. (**F**) Cathodoluminescence image of zircon rims around baddeleyite grains. Amp, amphibole; Bdl, baddeleyite; Mt, magnetite; Phl, phlogopite; Sulf, sulfide; Zrn, zircon.

A typical compound globule consists of a sulfide globule surrounded by a rounded halo with sharp margins (Fig. 1, A and B). These halos contain distinctly different mineral assemblages from the host mafic intrusion, which generally contains skeletal crystals of olivine, plagioclase, and chromian spinel (Fig. 2, A to C). With some minor variations, the compound globules are composed of clinopyroxene, plagioclase, phlogopite, ilmenite, apatite, amphibole, zircon/baddeleyite, calcite, and calcium hydrosilicates (*24*, *25*, *35*). Depending on the composition of the host mafic intrusion, the margin of the halo can be marked by a discontinuous chain of small euhedral and skeletal chromian spinel crystals rimming the outer margins of the halo (Figs. 1, A and B, and 2C). No chromian spinel occurs inside the halos.

**Fig. 3.**
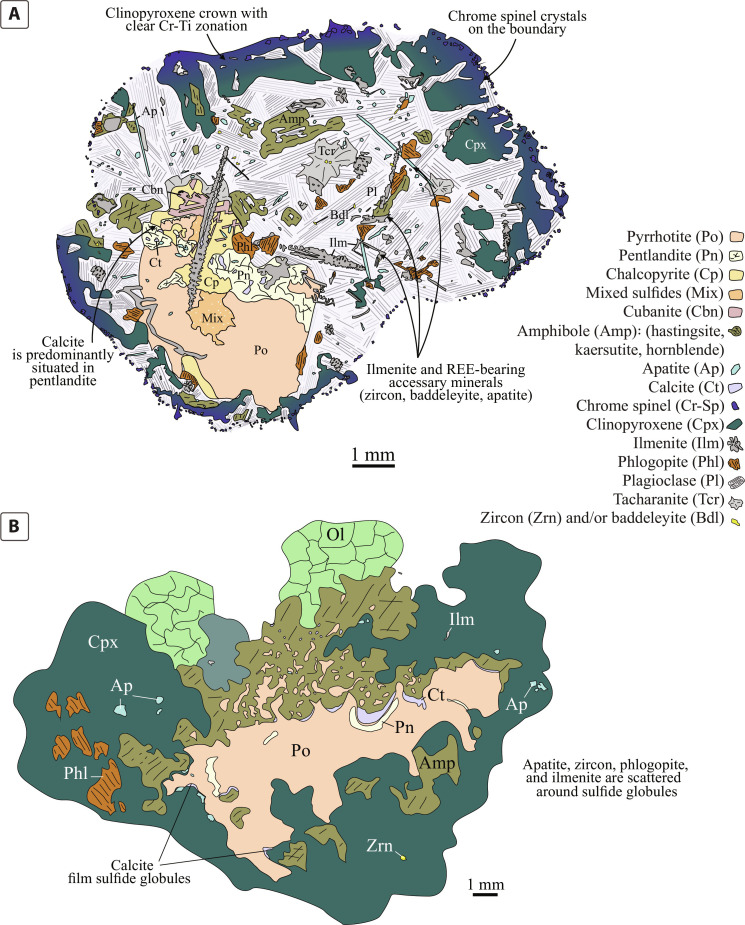
Schematic illustrations of two compound globules with similar mineralogical assemblages but different morphologies. (**A**) Mineralogy and morphology of a compound globule with a clear rounded halo from the upper crustal Rudniy intrusion, Mongolia (*36*). (**B**) An example of an obscure volatile- and incompatible element-rich halo from the lower crustal Valmaggia pipe, Italy (*29*, *37*).

The outer margin of the halos is marked by crowns of large (up to 5 mm) euhedral clinopyroxene crystals (Fig. 3A) growing inward toward the center of the halo. In most cases, the crystals terminate at the boundary, although they occasionally extend into the mafic host. Clinopyroxene and chromite crystals display chemical zoning with increasing Ti and decreasing Cr concentrations toward the centers of the compound globules. Most of the volume (about 60%) of the halos is occupied by plagioclase, euhedral amphibole (calcic hornblende and the sodic kaersutite and hastingsite), phlogopite, and other hydrous silicates, such as tacharanite [Ca_12_Al_2_Si_18_O_33_(OH)_36_]. Commonly, this assemblage includes apatite and ilmenite, which are generally preserved as skeletal elongated dendritic crystals (Fig. 2, D and E). Large skeletal crystals of ilmenite generally pierce sulfide globules, wrap around some portions of sulfide globules, or rim their edges (Fig. 1B).

Moreover, scattered grains of calcite, zircon, and baddeleyite are found within halos (Fig. 1, A to C). Calcite is found as rounded or irregular inclusions in sulfide minerals (Fig. 1E), predominantly in pentlandite, or as thin films on sulfide globule surfaces (Fig. 1, C and D). Baddeleyite, if present, is in contact with or as thin lamellae in ilmenite, or within rounded melt inclusions in ilmenite together with calcite and apatite. In some cases, both zircon and baddeleyite are present, either separately or together, where grains of baddeleyite are rimmed by zircon (Fig. 2F).

Ca-rich minerals, such as calcite, tacharanite, and Ca amphibole, are usually in direct contact with sulfide blebs. Rounded morphologies with sharp edges are occasionally not preserved around sulfide globules, yet the same mineral assemblages are always present in spatial association with sulfides (Figs. 1, C and D, and 3B). In these cases, volatile- and alkali-rich phases are dispersed around sulfides or form rims on globule surfaces (Fig. 1, C and D). Commonly, volatile- and incompatible element–rich minerals are found as rounded (Fig. 1E), vermicular (Fig. 1F), or irregularly shaped inclusions within sulfides.

### Carbonate coats rather than silicate caps

**Fig. 4.**
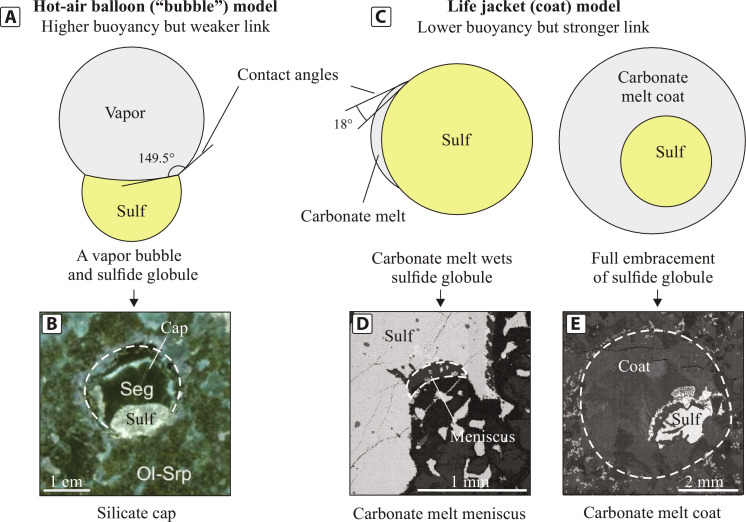
The differences in interfacial energies between pairs of sulfide liquid–vapor bubble and sulfide liquid–carbonate coat and resultant textural variations of sulfides associated with volatile-rich phases. (**A**) High interfacial energies between sulfide liquid and an attached vapor bubble result in obtuse interfacial contact angles (>90°) between the two. The resultant combination is buoyant due to the extremely low density of the vapor bubble but will only rise if the buoyancy forces overcome capillary forces. (**B**) Example of a silicate cap formed due to filling of a segregation vesicle (Seg) by late hydrothermal minerals (intergrowth of chlorite and serpentine) from the Black Swan komatiite-hosted deposit [modified after (*68*)]. (**C**) Low interfacial energies between sulfide liquid and a volatile- and incompatible element–rich carbonate melt result in wetting of the surface of the sulfide globule by carbonate melt or, if there is enough carbonate melt, complete coating of the sulfide globule. The resulting assemblage together with volatiles decreases the overall density relative to the host silicate melt. However, the link between sulfide globule and carbonate melt is stronger than that of sulfide globule and vapor bubble. The separation of this pair will be possible only if the carbonate melt is destabilized/dissolved into the surrounding silicate magma. (**D**) Carbonate meniscus filming a sulfide globule from the Valmaggia pipe, Italy. (**E**) Carbonate melt coat (made of clinopyroxene, plagioclase, calcite, amphibole, apatite, ilmenite, phlogopite, and zircon) from the Norilsk 1 intrusion, Russia. Ol-Srp, olivine pseudomorphosed by serpentine.

The distinctive rounded domains of volatile- and incompatible element–rich phases surrounding sulfide globules described in the Norilsk 1 intrusion have been previously denoted as silicate caps (*24*, *25*). This nomenclature aligns with the hypothesis that these domains represent remnants of vapor bubbles attached to the surface of a sulfide globule. This hypothesis was initially put forward based on experimental studies that demonstrated the strong affinity of vapor bubbles for sulfide globules (*21*). Originally confined to experimental studies and numerical modeling, only a few years later this compound droplet model was gradually applied to natural systems (*10*, *11*, *24*, *25*). According to this hypothesis, as magma ascends, the adherence of vapor bubbles to the surfaces of sulfide globules results in the formation of relatively buoyant compound droplets that resemble hot-air balloons transporting metal-rich sulfide cargos (Fig. 4A). Since these domains had rounded morphologies and contained minerals conventionally considered to be late or secondary, they were interpreted to represent the hollow spaces left behind by the dissipated vapor bubbles. Allegedly, these voids were later filled with highly fractionated silicate melt or secondary phases due to pressure-driven infiltration (*24*, *25*). This concept, initially deemed pressure independent (*27*), was further extrapolated to lower crustal levels (*10*, *11*). It was proposed that the adherence of supercritical CO_2_ liquid to sulfide globules might also play a pivotal role in extracting sulfide liquid from the mantle and facilitating its transport across the lithosphere. When a given intrusion crystallizes, it leaves behind sulfide minerals surrounded by carbonates, which usually film sulfide globules, as documented in the Valmaggia pipe, Italy (*37*).

Here, we argue that when this compound droplet model was extended from the metallurgical and experimental realms into the natural world, several crucial aspects were overlooked such that the model may have been overapplied across the entirety of magmatic sulfide systems, without consistently explaining the mineral assemblage and textures involved. For instance, apart from silicates, in many natural examples of compound globules oxides, phosphates and carbonates were documented (*10*, *37*–*39*). However, during interpretations, their presence was widely ignored. For example, the high modal abundance of calcite presents a conundrum to the theory that states that volatile- and incompatible element–rich domains formed due to the crystallization of a highly differentiated liquid, as carbonates are not stable in highly fractionated silicate magmas (*40*).

Most likely, calcite was not considered in previous interpretations because it was by default assumed to be secondary. However, its presence inside sulfide globules, the formation of vermicular textures, the lack of calcite veins, and the presence of thin films around sulfides instead of forming rims around calcium-bearing minerals confirm that calcite may be a primary magmatic phase. Moreover, the crystallization of calcite with skeletal dendritic crystals of ilmenite and apatite within coats would not occur during low-temperature late filling of hollow spaces. To develop such morphologies, they would have had to crystallize very rapidly, at the same time as dendritic crystals of olivine and spinel in the host mafic rock (Fig. 2).

However, even perfectly rounded halos surrounding sulfide globules do not mimic the textures reported in experiments. In experimental studies, gas bubbles are always observed attached to the upper side of sulfide globules (*19*, *24*), while in natural examples, except for subsurface and extrusive deposits, such as those associated with komatiites (Fig. 4B), volatile- and alkali-rich domains fully envelop sulfide globules (*35*). Since numerical modeling demonstrated that gas bubbles are unlikely to attain such exceptional wetting capabilities to fully embrace the sulfide globules (*21*, *26*, *27*), we propose that volatile- and incompatible element–rich halos formed from a liquid characterized by low interfacial energies in contact with sulfide liquid. In this case, the liquid wrapping a sulfide globule would act as a life jacket or coat that fully or partially isolates the sulfide globule from the silicate melt, in contrast to a cap-forming hot-air balloon (Fig. 4C). Therefore, we propose that a more reasonable name for those textures would be coats rather than caps. This distinction, between caps and coats, is not just semantic but is fundamental to the formation process, as they each represent distinct physical processes. Figure 4 illustrates this difference.

The bubble model explains this inconsistency between natural and experimental textures through two potential mechanisms: first, the change of relative surface tensions among vapor, silicate, and sulfide liquids, resulting in modified contact angles; second, the absorption of liquid sulfide inside the hollow spaces during pressure-driven infiltration of residual magmatic liquid or late secondary phases. However, no decompression experiments reproduced these vesicle fillings or textural modifications. Similarly in nature, there are cases where rapid crystallization or even quenching occurred in both silicate hosts and volatile-rich halos (e.g., in the deeper intrusive parts of the Norilsk magmatic complex and in the Rudniy intrusion), yet there are no examples of the presumed original textures of those alleged vapor bubble fillings with volatile- and alkali-rich minerals (*25*).

These observations raise doubts about the preservation potential of vapor bubbles within intrusive complexes. This is especially relevant at deeper levels in the crust due to the increasing solubility of CO_2_, H_2_O, and other volatiles with pressure (*41*–*44*). It was suggested that sulfide globules could be transported from metasomatized mantle domains by supercritical CO_2_ fluids (*10*). However, empirical evidence demonstrates that even in the unlikely event of magma reaching CO_2_ saturation at mantle or lower crustal depths, CO_2_ will not behave as a fluid but rather bond with Ca, K, Na, Mg, and Fe (which are abundant in the system) to form carbonate phases (*43*, *45*).

Considering the arguments above, we propose that the mineral assemblages surrounding sulfide globules reflect the presence of carbonate melts rather than vapor bubbles and therefore we will subsequently refer to them as “carbonate coats” (Fig. 4C). When compared to gas bubbles, carbonate melts are more stable over a much wider *P-T* range (*46*) and can more easily preserve their morphologies due to their lower interfacial energies at silicate melt contacts. Various textures emerge at this point, contingent upon the volume of carbonate melt present within the silicate liquid. When the volume is low, narrow menisci develop on the surface of sulfide globules, as illustrated in Fig. 4D. Conversely, a higher volume of carbonate melt facilitates the complete envelopment of the sulfide globule with a carbonate coat (Fig. 4E). The sharp changes in mineralogy within coats as well as the rounded shapes of the coats themselves could be explained by immiscibility between silicate liquid and the carbonate melt engulfing sulfide globules, as documented in the experiments discussed below.

Since traditional models of magmatic sulfide systems never incorporated carbonates, we conducted experiments to assess the likelihood of these three liquids coexisting at magmatic conditions. We chose the starting compositions of our experiments to reach three-liquid immiscibility. It is therefore important to note that our aim was not to reproduce specific magma compositions but rather to investigate the ability of carbonate melt to wet sulfides and form compound globules.

### Silicate–sulfide–carbonate melt immiscibility experiments

Numerous experimental studies have documented immiscibility between pairs of these melts—sulfide, silicate, and carbonate. However, although this three-liquid association has been previously documented in nature (*47*, *48*), only a few experimental studies have addressed the possibility of three immiscible phases coexisting (*49*). To test the hypothesis that the mineral assemblages coating sulfide blebs reflect an association with carbonate melt, we performed piston-cylinder experiments to assess the relationships between phases in a system containing these three liquids. Bulk starting compositions were roughly halfway between the binodal curves in immiscible silicate-carbonatite alkali systems [Table 1 (*50*)]. The experiments were conducted at a temperature of 1250°C and a pressure of 500 MPa within a 3-mm graphite internal capsule, surrounded by a 3.5-mm platinum external capsule. This platinum capsule was encased in a 5/8-inch MgO-Pyrex-NaCl assembly. Temperature measurement was facilitated using a B-type Pt-Rh thermocouple. Two experiments of different starting compositions were rapidly quenched from experimental conditions (*T* = 1250°C, *P* = 500 MPa), while a third experiment was slowly cooled to simulate textural evolution during natural crystallization (Table 1). Oxygen fugacity was buffered by coexisting graphite and in situ generated CO_2_ gas to conditions equivalent to roughly two log units below the fayalite-magnetite-quartz buffer (FMQ-2).

**Table 1.** Starting material compositions in experiments.

Exp 1				Exp 2 and 3			
Chemicals used for the starting composition mixture (mg)		Bulk chemical composition (wt %) of starting material		Chemicals used for the starting composition mixture (mg)		Bulk chemical composition (wt %) of starting material	
SiO_2_	133.7	SiO_2_	13.44	SiO_2_	55.58	SiO_2_	11.07
Al(OH)_3_	97.7	Al_2_O_3_	6.41	Al(OH)_3_	17.86	Al_2_O_3_	5.16
FeO(OH)	51.4	Fe_2_O_3_	4.64	FeO(OH)	33.74	Fe_2_O_3_	2.72
Mg_2_P_2_O_7_	51.4	MgO	5.57	Mg_2_P_2_O_7_	3.97	MgO	1.69
MgCO_3_	77.1	CaO	13.52	MgF_2_	10.92	Cr_2_O_3_	0.32
CaCO_3_	231.4	Na_2_O	4.83	Cr_2_O_3_	1.59	TiO_2_	1.38
CaSO_4_·2H_2_O	15.4	K_2_O	10.56	TiO_2_	6.95	CaO	19.94
Na_2_CO_3_	82.3	P_2_O_5_	3.29	CaCO_3_	178.64	Na_2_O	11.56
K_2_CO_3_	154.3	H_2_O	4.96	Na_2_CO_3_	97.26	K_2_O	2.69
FeS	77.1	CO_2_	22.62	NaCl	2.18	P_2_O_5_	0.50
NiSO_4_·6H_2_O	15.1	SO_3_	1.60	K_2_CO_3_	19.85	H_2_O	6.49
CuSO_4_·5H_2_O	12.9	FeS	7.74	FeS	11.91	CO_2_	24.95
		NiO	0.43	NiSO_4_·6H_2_O	29.77	SO_3_	3.71
		CuO	0.41	CuSO_4_·5H_2_O	29.77	F	1.33
						Cl	0.26
						NiO	1.69
						CuO	1.89
						FeS	2.37
Total	1000	Total	100	Total	500	Total	100

## RESULTS

**Fig. 5.**
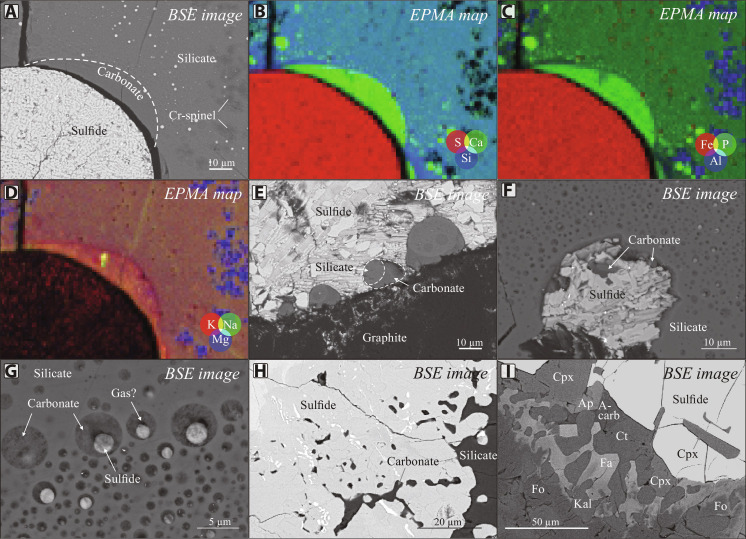
Photomicrographs of polished experimental capsules. (**A** to **D**) Resultant assemblage of Exp 1 (quenched experiment with high sulfide/carbonate ratio): (A) carbonate meniscus filming a large sulfide globule with a low contact angle, while the silicate glass contains small, dispersed sulfide globule–carbonate melt pairs (Exp 1); (B) the same globule showing false color RGB distributions of S, Ca, and Si; (C) Fe, P, and Al and (D) K, Na, and Mg. (**E**) Rounded inclusions of silicate-carbonate pairs in sulfide globules (Exp 1). (**F**) Large sulfide globule from Exp 2 (low sulfide/carbonate ratio experiment). The sulfide globule is rimmed by and includes carbonate. (**G**) Small carbonate-coated sulfide globules disseminated within silicate glass seen in (E) (Exp 2). (**H**) Vermicular and rounded inclusions of carbonate melt within a sulfide globule from Exp 3 (the slowly cooled experiment). (**I**) Absence of rounded carbonate coats around sulfides in Exp 3. A-carb, alkaline carbonate; Cr-spinel, chromian spinel; Fa, fayalite; Fo, forsterite; Kal, kalsilite.

The first quenched experiments (Exp 1, high sulfide/carbonate ratio) crystallized to an assemblage of several large sulfide globules distributed within a silicate matrix (Fig. 5, A and E). Carbonate melt was found only in contact with sulfide globules as relatively small menisci filming the sulfide globules (Fig. 5A) or as rounded inclusions within them. The contact angle between the carbonate melt menisci and large sulfide globules ranged from 26.5 to 32.6°. Qualitative electron probe microanalysis maps showing the major element distributions are provided in Fig. 5 (B to D). These distributions confirm the separation of three immiscible liquids, where carbonate melt is enriched in Ca, K, Na, and P; silicate melt is enriched in Mg, Al, and Si, and sulfide melt contains S and Fe. Several large sulfide domains within the silicate matrix contained carbonate-silicate melt pairs as inclusions rather than films (Fig. 5E). In this case, carbonate melt would always have lower wetting angles than silicate spheres in contact with sulfides.

**Fig. 6.**
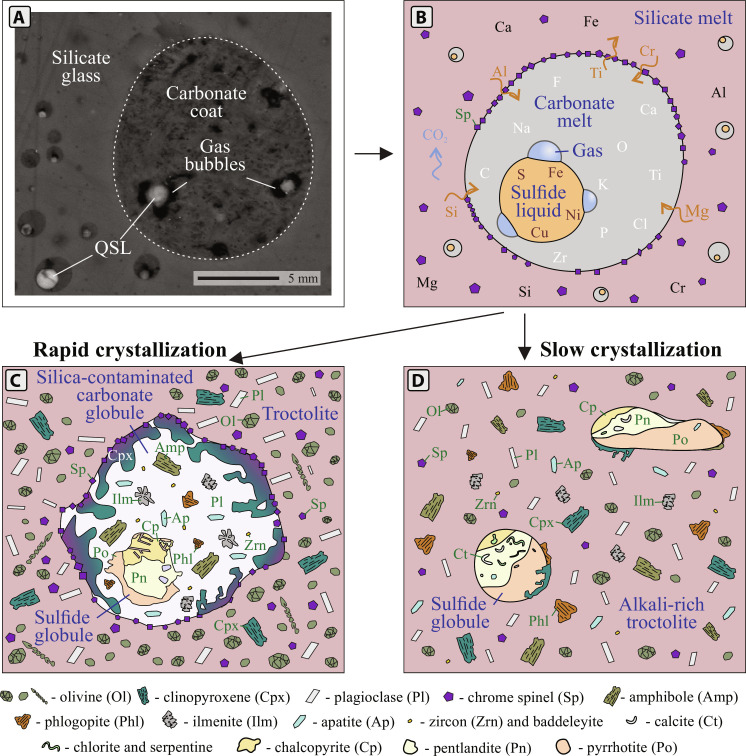
A schematic illustration of the processes occurring during crystallization of alkali-rich mafic magmas based on experimental results and natural examples. (**A**) BSE image of immiscible compound sulfide–carbonate blebs containing vapor bubbles from Exp 2. (**B**) Separation of alkali-rich magma into three immiscible liquids—silicate, carbonate, and sulfide. Sulfide and carbonate liquids wet each other forming compound globules. During ascent, carbonate melt exsolves CO_2_, making compound globules more buoyant. (**C**) Carbonate-sulfide compound globule in mafic magma that underwent rapid crystallization. (**D**) The case of slow crystallization, carbonate melt does not preserve globular shapes, and alkali-rich minerals surround sulfide globules. QSL, quenched sulfide liquid.

The silicate glass in the second experiment (Exp 2, low sulfide/carbonate ratio) also contained large sulfide globules with films and inclusions of carbonate melt (Fig. 5F) and numerous scattered small (0.5 to 15 μm in radius) compound globules of sulfides coated by carbonate (Fig. 5G). The resulting compositions of sulfide, silicate, and carbonate glasses are provided in Table 2. Most of these small globules included rounded dimples that most likely represent voids left by gas bubbles (Figs. 5G and 6A). As predicted by traditional vapor bubble–sulfide liquid experiments, gas and sulfide stick to each other (*21*, *26*). But here they are found only within carbonate-sulfide compound globules, not in the silicate glass.

**Table 2.** The qualitative average compositions of silicate, sulfide, and carbonate liquids in the second experiment (in at %). The data are obtained using EDS. *n*, number of analyses.

	Carbonate	Silicate	Sulfide
*n*	15	18	6
O	42.72	41.19	8.04
F	4.74	2.30	
Na	16.15	8.66	1.24
Mg	1.37	3.74	
Al	0.58	5.34	
Si	1.56	15.21	
P	1.32	0.40	
S	0.72	0.37	32.08
Cl	0.70	0.50	
K	2.52	1.24	0.50
Ca	27.29	19.11	0.24
Ti	0.41	1.86	
Cr		0.22	0.89
Fe	0.88	0.57	33.61
Ni			22.50
Cu			6.27

The third, slowly cooled experiment (Exp 3), contained several medium to large sulfide globules. Rounded and vermicular carbonate inclusions (Fig. 5H) are common within sulfide globules. The matrix contains chromian spinel, nepheline, apatite, Na–K–Ca-carbonate, and clinopyroxene. There is no clear spatial separation between silicate and carbonate minerals, indicating that while compound globules formed at peak conditions, they were not preserved during slow cooling (Fig. 5I). A schematic illustration of the experimental results at different crystallization rates is provided in Fig. 6 (B to D).

## DISCUSSION

### The spatial relationships between three immiscible liquids—carbonate, sulfide, and silicate

**Fig. 7.**
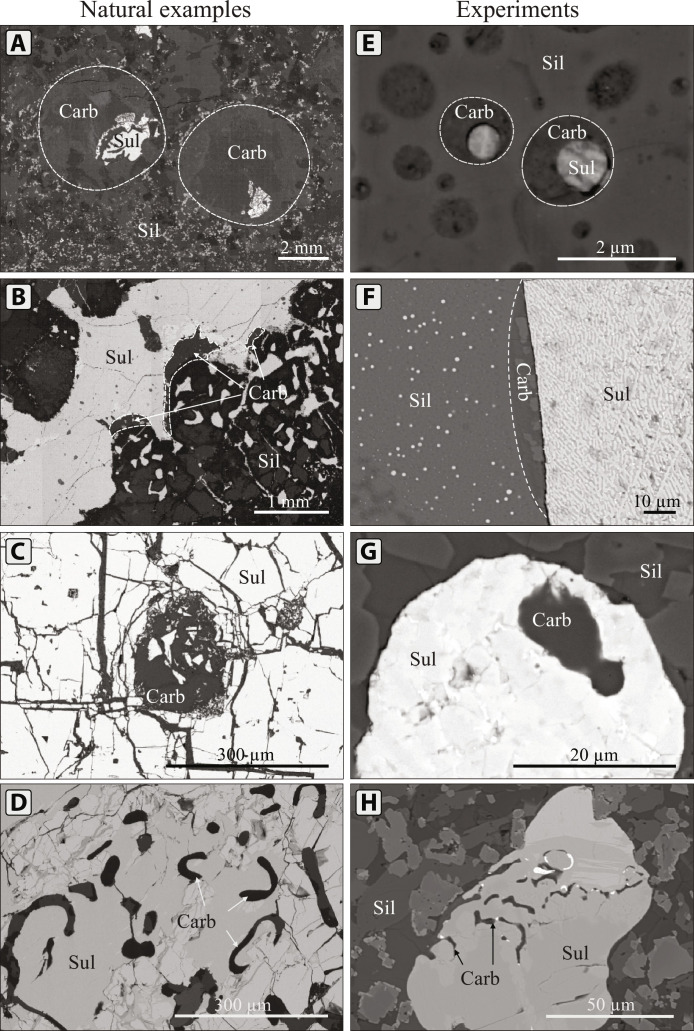
BSE images of textural relationships between carbonate, sulfide, and silicate melts from natural (left column) and experimental examples (right column). (**A**) Carbonate-sulfide compound globules from the Norilsk 1 intrusion, Russia. Described in (*35*). (**B**) Carbonate films on the surface of a sulfide globule from the Valmaggia pipe, Italy. (**C**) Rounded calcite inclusion in a sulfide globule from the Rudniy intrusion, Mongolia. (**D**) Vermicular inclusions of calcite, apatite, chlorite, and serpentine in a sulfide globule from the Rudniy intrusion. (**E**) Carbonate filming sulfide globules in Exp 2. (**F**) Carbonate melt coating sulfide globules in Exp 1. (**G**) Rounded inclusion of carbonate melt in a sulfide globule in Exp 3. (**H**) Vermicular inclusions of carbonate melt in Exp 3. Carb, carbonatite; Sil, silicate; Sul, sulfide.

Similarities between experimental and natural carbonate-sulfide-silicate textures are summarized in Fig. 7. The textures of carbonate melt–sulfide interactions generated during the experiments perfectly reproduce those found in nature. The rounded shapes of compound globules reflect three liquid immiscibility (carbonate, sulfide, and silicate melts) and preferential adherence of carbonate melt to sulfides at run conditions. In the first experiment, the acute contact angle confirms strong adhesive forces and low surface tension between the two liquids, enabling the carbonate melt to wet sulfides and spread over their surfaces (Fig. 7, B and F). The prevalence of adhesive forces in this case is distinct from any sulfide globule–bubble experiments (*21*).The low surface tension between the carbonate melt and sulfide globules leads to complete envelopment of the sulfide globules within carbonate melt once a lower sulfide/carbonate ratio is attained, as shown in the results from Exp 2 (Fig. 7, A and E).

Note that rounded carbonate morphologies were not preserved in slowly cooled experiments (Fig. 5I). At progressively lower temperatures, carbonate melt starts crystallizing into solid phases and globular textures disappear. Since separation of the immiscible carbonate melt from silicate liquid is unlikely due to low interfacial energies between the two liquids (*51*), we propose that globular morphologies are not usually preserved in nature because immiscibility of carbonate melt is destabilized during cooling and differentiation, or that minerals nucleate within one liquid and grow through the other, overprinting original globular textures. Carbonate–sulfide melt interactions also explain the presence of textures at odds with the previous (bubble) concept, such as inclusions of volatile-rich phases within sulfides (Fig. 7, C and G).

### Silica contamination of carbonate melt globules

The compositions of our experiments were selected to be strongly alkaline to assure experimentally accessible immiscibility and permit investigation of the physical properties of the three liquids. However, mineralized zones in both the Rudniy and the Norilsk intrusions are associated with low-alkali tholeiitic basalts. Although the miscibility gap between low-alkali mafic silicate melts and carbonate liquids is narrow, we cannot completely rule out the possibility of magmas evolving to high alkali contents during ascent, fractionation, and crystallization. It has been noted that less alkali-rich compositions can also lead to silicate-carbonate immiscibility (*50*), resulting in crystallization of calcite and plagioclase, with lower alkali contents sequestered into phlogopite and mildly alkali amphiboles such as kaersutite, as observed in our samples. Intensive fractionation or addition of alkalis and CO_2_ from the mantle source or contamination could theoretically shift the composition of the silicate melt into the miscibility gap.

Mineral assemblages coating sulfide globules in the natural examples described here and elsewhere (*24*) are dominated by silicate minerals, hence their common description as “silicate caps.” It is well known that carbonate melts typically contain very low silica concentrations (*52*). If we argue that this assemblage represents crystallization from carbonate melt, it is important to clarify the differences between conventional compositions of carbonate melts and the carbonate coats documented here, which have high silica and alumina contents resulting in an unusual abundance of silicates and a high concentration of TiO_2_, reflected in the presence of multiple ilmenite inclusions. Moreover, the dominant carbonate in our experiments is nyerereite (Na,K)_2_,Ca(CO_3_)_2_ or a compositionally related phase. However, alkali carbonates are not present in natural samples, which instead contain abundant plagioclase, an atypical mineral for carbonatites (*53*).

Two scenarios could be envisaged to explain these observed inconsistencies. First, the initial carbonate melt might have had a slightly different initial composition due to unusual conditions and equilibrium with sulfide liquid and mafic silicate melt, an area widely understudied. Second, the resulting assemblage could form due to late-stage exchange reactions between silicate and immiscible carbonate melts. Many parameters control CO_2_ solubility in silicate magma or silica contents of carbonate melt. For example, the addition of water substantially increases the width of the miscibility gap between silicate and carbonate liquids (*54*). Also, at the high temperatures (>1200°C), typical of magmatic sulfide systems, carbonate melt can dissolve up to a few wt % of SiO_2_ and Al_2_O_3_ (*50*).

Carbonate melt–sulfide compound globules are hosted in high-Mg silicate magmas, where one of the first minerals to crystallize is olivine. Crystallization of olivine drives silica contents and activities higher, leading to disequilibrium between the evolved silicate liquid and carbonate melt. Other evidence for increasing silica activity and contamination of the carbonate melt is the coexistence of zircon and baddeleyite and the formation of clinopyroxene crowns around coats enveloping the sulfide globules.

In silica undersaturated carbonate melts, the stable zirconium mineral is baddeleyite (ZrO_2_), while zircon (ZrSiO_4_) requires higher silica activity and thus only forms in contaminated carbonate melts (*55*). In the natural examples studied here, zircon coexists with baddeleyite (in some cases forming rims around baddeleyite grains), a very unlikely pairing given the poor silica activity buffering capacity of the miniscule ZrO_2_ contents in the system. Most likely, their coexistence indicates the incomplete reaction ZrO_2_ + SiO_2_ = ZrSiO_4_ in a regime of increasing silica activity.

Another argument supporting the reaction between carbonate and silicate melts is the presence of clinopyroxene crowns rimming carbonate coats. Clinopyroxene is ubiquitous at silicate–carbonate melt interfaces during silica contamination (*53*) and was also documented in mantle xenoliths (*56*). Experiments show that clinopyroxene formed in this way is zoned, with compositional domains reflecting the medium from which it crystallized (*57*). These features match our observations, with the Cr-rich clinopyroxene zones on the spinel-bearing silicate side and the clinopyroxene Ti-rich zones on the ilmenite-bearing carbonate side. We suggest that most, if not all, silicate minerals within coats are the result of silica contamination. This process also explains the lack of dolomite and other carbonates in the mineral assemblages, as the magnesian, potassium, and sodium components are instead partitioned into silicate phases (*53*).

### Source of carbonate melt

Carbonatites originate from various sources and have complex evolutionary records. However, it has been unequivocally shown that carbonatite can originate from mantle-derived melts (*58*). The exact mechanism behind the interaction of carbonate melts with ultramafic magmas is still a matter of debate. Some researchers argue that carbonatite melts can form during partial melting of carbonated peridotite and easily migrate through silicate grain boundaries as incipient melts, eventually accumulating relatively large volumes of carbonatite (*59*). Others suggest that carbonatites form together with silicate melt via liquid immiscibility with sequential separation of carbonatite due to crystal fractionation (*50*). It was also demonstrated that the formation of carbonatites could be related to plume activity (*60*).

Both the Rudniy and the Norilsk intrusions are associated with plume-related magmatism (*61*–*63*). It is likely that carbonate and silicate melts could interact within the mantle-to-crust magmatic plumbing network. There is no reason to assume that only one unique mechanism can be applied to all mafic and carbonate complexes worldwide as little is known about the processes that promote melt migration through the lithosphere. It is therefore possible that immiscibility between mafic silicate and carbonate melts occurred at some stage when magma reached the right conditions/composition, leaving behind little to no petrological evidence of this process.

However, the droplets of carbonate melt documented in this study are unlike the well-known carbonatites from alkali-rich complexes. These melts are immiscible carbonate droplets that form when sufficient alkalis and CO_2_ are present to stabilize carbonate melt. Thus, the carbonate melt droplets shown here are not in any way, suggested to be the source of carbonatites, sensu stricto. Here, tiny droplets destabilize during cooling to form silicate-rich coats with occasional calcite that survives decarbonation.

### Metal transport

In the model presented here, metal-rich sulfide liquids exsolved from basaltic magma wear coats of carbonate melt, which isolate each droplet from the surrounding silicate magma and solid phases (Fig. 4C). Carbonate melts are extremely mobile due to their low viscosity (*64*), low density (≤2.7 g/cm^3^) (*65*), exceptional wetting capabilities, and higher melt-solid interfacial energy than silicate liquid (*51*). It seems that sulfide globule size matters for the transport of metal-rich sulfide cargos. The fact that relatively large sulfide globules are never fully surrounded by carbonate liquid may indicate that the sulfide/carbonate ratio should be relatively low to overcome the density contrast problem.

A light gaseous bubble would behave as a hot-air balloon dragging its heavy sulfide cargo upward [Fig. 4A (*21*, *27*)]. However, the physical link between the pair will be quite tenuous when it traverses crystal mush containing grains of variable size and morphology (*16*). Conversely, a coat composed of a relatively buoyant liquid may act more as a life jacket, decreasing the density contrast between sulfide and silicate liquid and isolating sulfide globules from the surrounding silicate crystal-melt mixture. The presence of carbonate melt coating the sulfide globule rather than a gaseous bubble dragging it upward would also have the advantage of effectively isolating it from the surrounding silicate magma, slowing down its dissolution upon ascent. As a result, this newly recognized process identifies an important change in the way we understand metal flux associated with sulfide transport across the lithosphere. However, we acknowledge that this may not operate everywhere, and therefore, it may not be the only process at play in the genesis of magmatic sulfide systems.

The addition of H_2_O and Cl, as reflected in the abundance of hydrous (amphibole, phlogopite, tacharanite) and Cl-rich minerals (apatite) associated with magmatic sulfides, further decreases the density of the carbonate melt, increasing its buoyancy and mobility. Furthermore, gas bubbles, which mostly adhere to sulfide liquid in our experiments and elsewhere [Figs. 5G and 6A, (*21*, *26*)], most likely contain CO_2_ and H_2_O, either formed by decarbonation reactions or scavenged from the decompressing silicate melt (*66*). Decarbonation reactions proceed by introduction of lighter elements, such as SiO_2_ and Al_2_O_3_ from the silicate liquid, diluting heavier elements and further reducing average densities of compound globules. In both scenarios, the addition of CO_2_ bubbles into the compound globules would decrease density, thereby increasing mobility within the silicate melt. The role of CO_2_ gas increases as both pressure and CO_2_ solubility decrease. The average density of a carbonate melt–sulfide compound globule that includes volatiles would roughly be equivalent to the density of mafic silicate melt, effectively neutralizing the density contrast between sulfide and silicate liquids. The scenario outlined here, which we tested experimentally, presents an efficient pathway for metal transport from mantle to crust.

The interfacial energies between melt and solid phases of carbonate, sulfide, and silicate melts are crucial in determining the behavior of metal-rich sulfide liquids within silicate magmas. Among these three liquids, carbonate melt has the highest solid-melt interfacial energies, which it minimizes by reducing contact with solid phases. As a result, carbonate melt ocelli tend to avoid any interaction with crystal mush, limiting their contact with solid phases in magmas (*51*) and isolating sulfide globules from solid phases. In other words, carbonate acts as a lubricant for sulfide. As a result, sulfide liquid can remain mobile, allowing for its transport to higher levels in the crust.

Accordingly, the carbonate-coated metal-rich cargo could be transported with the magma across the lithosphere, thereby increasing the probability of forming sulfide deposits when magma was dynamically concentrated into structural traps along the plumbing network. Alternatively, the metal budget associated with mantle-derived coated sulfides could enhance the fertility of mineralized magmas that become sulfide-saturated during crustal contamination at various crustal levels. The final mineralized product would then be composed of multiple sulfide populations of different origins displaying variable physiochemical and isotopic characteristics (*67*), reflecting the presence of deeply sourced transported sulfides intercalated with sulfides sourced locally. The two processes are not mutually exclusive, but further investigation of the source of carbonate melt in magmatic sulfide systems is required.

These diverse scenarios give rise to a broad spectrum of textures and assemblages that are commonly observed in natural occurrences. The specific textures and assemblages depend on factors such as pressure, temperature, fluid content, and the rate at which crystallization occurs. It is intriguing to note that we can only observe these distinctive rounded textures as a result of rapid cooling, which allows these textures to solidify before they become destabilized (Fig. 6C). In most cases, the sole indication of the initial presence of carbonate melt is sporadically dispersed alkali- and volatile-bearing phases within alkali-rich mafic rock that host sulfide globules (Fig. 6D).

In summary, although we acknowledge that multiple processes may be involved in metal sequestration from the mantle and transport to the upper crust, it seems likely that large deposits may occur only when multiple processes operate simultaneously. The carbonate melt hypothesis proposed in this study addresses a number of the shortcomings that affected previous models. An improved understanding of the geological processes related to sulfide melt transport in mafic magmas will underpin advances in the exploration, characterization, and processing of critical metal resources for the future.

## MATERIALS AND METHODS

Mineralogical, textural, and compositional characteristics of the samples from the Rudniy and Norilsk 1 intrusions, as well as the Valmaggia pipe have been studied in polished thin sections using optical microscope Zeiss (transmitted and reflected light), scanning electron microscope (SEM) Tescan Vega 3 with energy-dispersive spectroscopy (EDS) (analytical conditions of 20-kV accelerating voltage, 15-nA beam current, 10-s counting time for point analysis), and Tescan integrated mineral analyzer (TIMA) SEM (accelerating voltage 25 kV, probe current 7 nA, beam intensity 20 nA, pixel size 3 μm).

Experiments were carried out at the Australian National University (ANU), using a piston-cylinder apparatus. Starting materials were prepared by mixing the reagents listed in Table 1 using an agate mortar and pestle in acetone. The experiments were run at 1250°C and 500 MPa in a 3-mm graphite internal capsule enclosed by a 3.5-mm platinum external capsule. The platinum capsule was enveloped in a 5/8-inch MgO-Pyrex-NaCl assembly. Temperature was monitored using a B-type Pt-Rh thermocouple. Constant temperature experiments (Exp 1, D3212; Exp 2 D3375) were run on manual piston cylinders. After an initial pressure increase of about 1 kbar for electrical contact, the temperature was increased at 150°C/min, while pressure was manually increased simultaneously to run conditions. Exp 3 (D3374) was conducted on an automated piston cylinder with pressure and temperature controlled automatically using in-house software. Pressure was increased over 5 min and temperature to 1300°C over 25 min.

The first experiment (Exp 1) was quenched after dwelling 4 hours at 1250°C, the second experiment (Exp 2) was quenched after dwelling 19 hours at 1250°C, and experiment 3 (Exp 3) was steadily cooled down over 19 hours to 300°C before quenching. During quench, the temperature reached room temperature in under 20 s, with a steep temperature decrease of several hundreds of degrees achieved within the first few seconds. Oxygen fugacity was buffered to near the carbon-CO_2_-CO oxygen buffer (roughly FMQ-2 at our run conditions), using an in situ reaction between oxidized sulfate starting materials and graphite. During the run, base metal sulfates were reduced to base metal sulfides, generating CO_2_ in excess of carbonate-bound CO_2_. Thus, the coexistence of graphite and free CO_2_ vapor served as the oxygen buffer. Minor dilution of CO_2_ in the vapor by H_2_O shifts buffered *f*o_2_ (oxygen fugacity) to lower values by a negligible amount. After each experiment, the capsules were mounted in epoxy resin and exposed using sandpaper by dry polishing owing to the water solubility of some experimental products. Further reimpregnation with epoxy resin was conducted to mechanically stabilize the materials for subsequent polishing stages. Final polishing was conducted using diamond paste down to 0.25 μm for observation. The resulting assemblages were manually polished and studied using SEM with EDS at the Centre for Microscopy, Characterisation and Analysis, Perth, Australia. Specifically, a Tescan Vega 3 SEM equipped with EDS and a FEI Verios 460 SEM with an Oxford X-Max 80 silicon drift x-ray spectrometer (EDS) were used. For the mapping of the experimental assemblages, a JEOL JXA-8530F Plus electron probe microanalyzer (Centre for Advanced Microscopy at the ANU) was used (accelerating voltage 15 kV, probe current 10 nA, beam intensity 15 nA, pixel size 1 μm).
